# Initial Experience after Transition to Immune Checkpoint Inhibitors in Patients with Non-small Cell Lung Cancer Treated in a Rural Healthcare Region

**DOI:** 10.7759/cureus.7030

**Published:** 2020-02-18

**Authors:** Carsten Nieder, Anne Reigstad, Espen A Carlsen, Liv Flatøy, Terje Tollåli

**Affiliations:** 1 Oncology, Nordland Hospital Trust, Bodø, NOR; 2 Internal Medicine, Nordland Hospital Trust, Bodø, NOR

**Keywords:** non-small cell lung cancer, systemic therapy, chemotherapy, immune checkpoint inhibitor

## Abstract

Objective

The aim of this study was to investigate the patterns of palliative care, terminal care, and hospital deaths in deceased patients with non-small cell lung cancer (NSCLC) treated with immune checkpoint inhibitors (ICI).

Methods

This study involves a retrospective analysis of a group of 32 patients treated with first- or second-line ICI regimens. The group was compared with a matched contemporary cohort of patients who received systemic treatment that did not include an ICI. The 1:1 matching was based on sex, age, stage of cancer (IV versus lower), and initial treatment after diagnosis (locoregional versus systemic).

Results

The median overall survival from diagnosis was 9.8 months [95% confidence interval (CI): 7.4-12.2 months] in the non-ICI patients and 11.6 months (95% CI: 5.9-17.3 months) in the ICI group (p: 0.09). Death resulting from toxicity was recorded in two patients (non-ICI) and one patient (ICI), respectively (p: 0.8). Hospital death was more common after ICI (19 versus 11 patients, p: 0.08). During the last three months of life, non-ICI patients spent a median of 11 days (range: 0-28) in the hospital, compared with 20 days (range: 0-45) for ICI patients (p: 0.005). More ICI patients (21 versus 14) received systemic therapy during the last three months of life (p: 0.13). However, treatment rates during the last four weeks were comparable (eight non-ICI and six ICI patients, respectively; p: 0.8).

Conclusion

We did not identify any concerns regarding the fatal toxicity of ICI treatment. Due to several different baseline parameters, there are reasons to believe that hospitalization and hospital death in the ICI group were mainly related to unevenly distributed disease characteristics and not to ICI administration itself. Since real-world data from rural patient cohorts might differ from those obtained in clinical trials, it is necessary to conduct additional and larger studies about ICI-associated patterns of terminal care.

## Introduction

The systemic treatment of advanced non-small cell lung cancer (NSCLC) has recently undergone significant transformations [1,2]. Platinum-based first-line chemotherapy and previous second-line regimens have been replaced by treatment with immune checkpoint inhibitors (ICI) such as pembrolizumab, atezolizumab, and nivolumab, both as monotherapy in first- or second-line treatments or in combination with chemotherapy in first-line treatment [3-7]. For some combinations, a specific histology or programmed cell death ligand (PD-L1) expression is required [8]. In Norway, the national lung cancer group (NLCG) and the governmental commission for approval and remuneration of new drugs have sequentially introduced monotherapy with pembrolizumab in first-line treatment for patients with high PD-L1 expression, monotherapy with atezolizumab in second-line for patients with PD-L1 positive tumors, and combined pembrolizumab/platinum/pemetrexed in first-line for patients with non-squamous NSCLC. Traditionally, overly aggressive end-of-life (EOL) care has been identified as one of several challenges in the treatment of incurable NSCLC [9]. Together with several other groups from various countries, we have previously examined the patterns of palliative care, terminal care, and hospital death in patients with NSCLC [10-12]. Therefore, we were interested in exploring potential changes in such quality-of-care indicators in the transition phase during the early adoption of ICI treatment for NSCLC. Based on those considerations, the present retrospective quality-of-care study was performed.

## Materials and methods

This study included all patients who had died from NSCLC in the catchment area of the Nordland Hospital Trust (NHT), Bodø after having received at least one cycle of ICI therapy. In this geographical region (population: approximately 150,000), all cancer care is prescribed, supervised, and guided by the oncology department at NHT. NHT is owned by the Ministry of Health and Care Services and administered through a regional trust (North Norway Regional Health Authority trust; www.helse-nord.no). Private pulmonology or oncology services are not available in our healthcare region. This fact and the structure of the publicly-funded national healthcare system facilitate analyses of unselected cohorts, which resemble population-based cancer registries. However, cancer registries include much larger patient cohorts.

The electronic patient records (EPR) of NHT were used to identify all eligible patients, i.e., those treated for histologically confirmed NSCLC. For this study, patients who had died from their disease during the time period from January 1, 2016 to December 31, 2019 were selected. The initial diagnosis of NSCLC could have been made before 2016. Complete medical records, including death certificates and baseline demographic data, were available in the hospital's EPR system. All information was reviewed retrospectively, starting from the first referral for suspected lung cancer until patients' death. All patients in this study had been covered by the Norwegian public healthcare system, which pays for diagnostic tests, treatment, hospitalization, follow-up care, travel, and accommodation. As a result, no financial barriers had prevented access to ICI therapy and hospital care for these patients. All lung cancer treatment had been in accordance with Norway's national guidelines. Therapeutic pathways were developed by the NLCG (www.nlcg.no) and guided by the decisions made by a multidisciplinary lung tumor board, which meets at NHT on a weekly basis. Guideline-based routine oncology care included systemic cytotoxic chemotherapy, tyrosine kinase inhibitors (if the appropriate target was present), ICI, radiation therapy, thoracic surgery, and supportive/palliative measures. None of the patients had participated in a prospective clinical study or expanded access program. In other words, an ICI was prescribed only after its approval in Norway, as part of NLCG recommended care, and in cases that met Eastern Cooperative Oncology Group (ECOG) performance status (PS) of 0-2. 

Eligible patients were selected from the above-mentioned EPR system, and the IBM SPSS version 25 software package (IBM, Armonk, NY) was employed for all statistical analyses. We used the preexisting and continuously updated database to create a matched contemporary cohort of patients who received systemic treatment that did not include an ICI [10-12]. The main reasons for not receiving an ICI had been lack of availability, ongoing corticosteroid treatment, and autoimmune comorbidity. The 1:1 matching was based on sex, age, cancer stage (IV versus lower), and initial treatment after diagnosis (locoregional versus systemic). For comparison of dichotomous variables, the Chi-square test and Fisher’s exact test, where applicable, were employed. For continuous variables, the Mann Whitney U test was employed. The significance level was set to 5% and all tests were carried out two-sided. The Kaplan-Meier method was used to analyze actuarial overall survival from the imaging diagnosis of lung cancer. Censoring was not necessary as all patients were deceased at the time of this analysis. Survival differences were compared with the log-rank test. The study was performed as a retrospective analysis of EOL care for NSCLC. Since this was a quality-of-care analysis, no approval from the Regional Committee for Medical and Health Research Ethics (REK) was necessary.

## Results

The study included 32 deceased patients, of whom 21 had received second-line ICI therapy after previous platinum-based chemotherapy (all PD-L1 positive, mostly atezolizumab). Eight patients had received first-line ICI monotherapy with pembrolizumab (all PD-L1 positive with at least 50% expression). The remaining three patients had received first-line pembrolizumab/carboplatin/pemetrexed (all non-squamous cell cancer, PD-L1 negative or expression <50%). Previous targeted therapy had not been given, because targetable mutations or molecular features had been absent. Table 1 shows the patient characteristics for the ICI group and the matched group of 32 comparable patients managed without ICI during the same time period.

**Table 1:** Baseline characteristics of patients at diagnosis of lung cancer ICI: immune checkpoint inhibitor; T: the size and position of the tumor; N: the presence of spread into the lymph nodes *E.g., endobronchial stent or laser treatment

Characteristics	Non-ICI	Non-ICI	ICI	ICI	P-value
Number of patients	32	100%	32	100%	
Age in years, median (range)	69 (55-77)		69 (51-81)		>0.3
Female	16	50%	16	50%	>0.3
Male	16	50%	16	50%	
Married or partnered	23	72%	21	66%	>0.3
Single	9	28%	11	34%	
No comorbidity	11	34%	14	44%	>0.3
Cardiovascular comorbidity	11	34%	5	16%	
Diabetes mellitus	10	31%	7	22%	
Chronic obstructive pulmonary disease	0	0%	3	9%	
Active smoker	14	44%	7	22%	0.11
Weight loss of at least 5%	15	47%	16	50%	>0.3
Squamous cell cancer	8	25%	14	44%	0.19
Non-squamous cell cancer	24	75%	18	56%	
T1-2	20	63%	11	34%	0.04
T3-4	12	38%	21	66%	
N0-1	8	25%	10	31%	>0.3
N2-3	24	75%	22	69%	
Stage I or II	1	3%	2	6%	>0.3
Stage III	9	28%	8	25%	
Stage IV	22	69%	22	69%	
Liver metastases	4	13%	7	22%	>0.3
Bone metastases	5	16%	8	25%	>0.3
Brain metastases	8	25%	5	16%	>0.3
Pleural or contralateral lung metastases	8	25%	11	34%	>0.3
Curative intent in first-line	3	9%	7	22%	0.3
Any palliative care team involvement	19	59%	16	50%	>0.3
Any thoracic radiotherapy	18	56%	22	69%	>0.3
Only one line of systemic therapy	19	59%	8	25%	0.13
Two lines of systemic therapy	11	34%	15	47%	
More than two lines of systemic therapy	2	6%	9	28%	
Intervention during the last month*	0	0%	0	0%	>0.3
Pleurodesis or drainage (last month)	3	9%	10	31%	0.06
Death expected, therapy ceased	17	53%	15	47%	>0.3
Unexpected death, still on therapy	8	25%	8	25%	
Medical records lack sufficient details regarding this parameter	7	22%	9	28%	

Differences between the two groups were noted for several parameters including T stage (p: 0.04, in favor of non-ICI patients), active smoking (p: 0.11, not significant but in favor of ICI patients) and therapy with more than one line of systemic treatment (p: 0.008, in favor of ICI patients).

The median overall survival from diagnosis was 9.8 months [95% confidence interval (CI): 7.4-12.2 months) in the non-ICI patients and 11.6 months (95% CI: 5.9-17.3 months) in the ICI group (p: 0.09). Death resulting from toxicity was recorded in two patients (non-ICI) and one patient (ICI), respectively (p: 0.8). Hospital death was more common after ICI (19 versus 11 patients, p: 0.08, not significant). During the last three months of life, non-ICI patients spent a median of 11 days (range: 0-28) in hospital, compared with 20 days (range: 0-45) for ICI patients (p: 0.005). Only one patient in each group was never hospitalized during the last three months of life. The main reasons for hospitalization were cancer-related symptoms and infections, rather than side effects of treatment. More ICI patients (21 versus 14) received systemic therapy during the last three months of life (p: 0.13, not significant). However, treatment rates during the last four weeks were comparable (eight non-ICI and six ICI patients, respectively, p: 0.8).

The median overall survival from diagnosis was 8.7 months (95% CI: 3.3-14.1 months) in 11 patients treated with first-line ICI with mono- or combination therapy, as compared to 14.7 months (95% CI: 6.6-22.8 months) in 21 patients treated with second-line ICI monotherapy. 

## Discussion

This study evaluated the patterns of care in the first patients treated with ICI regimens for NSCLC in our health care region in rural Norway. Monitoring of new treatment paradigms is paramount because real-world patients often differ from their counterparts who were included in prospective clinical trials with stringent eligibility criteria [13]. We focused on deceased patients (n = 32) rather than patients currently doing well while still on treatment or not receiving any subsequent therapy (n = 37, 54% of all patients with ICI therapy for NSCLC). The latter group was slightly larger and included some patients with more than two years of follow-up after starting with ICI. Therefore, the survival results underestimate the true median survival and are not representative of ICI treatment in general. We did not include patients managed with the PACIFIC trial regimen (durvalumab after chemoradiation), which has replaced previous chemoradiation approaches for non-metastatic disease [14,15]. A typical patient in the present study had non-squamous stage IV disease and was older than 65 years of age, as displayed in Table 1. The main limitation of this study was the small number of patients and, consequently, limited statistical power. It was not possible to match the non-ICI cohort with regard to all potential prognostic factors. Resulting imbalances included those relating to the T stage. However, sex, age, overall stage, and primary treatment strategy were comparable between both groups.

We did not identify any concerns regarding the fatal toxicity of ICI treatment. However, ICI patients spent a significantly longer time in the hospital compared to non-ICI patients during the final three months of life. Apparently, this was not caused by ICI toxicity. Interestingly, ICI patients received pleurodesis and drainage more often than non-ICI patients. Likely, this reflects the different stages of thoracic disease, as more ICI patients had T3-4 tumors and pleural or contralateral lung metastases (ipsilateral lung metastases are covered in the T classification). Importantly, the staging was performed at diagnosis. Possible changes in the terminal phase of the disease have not been evaluated in this study. 

In a previous study (pre-ICI era), 29% of the patients in our region had received oncological non-ICI treatment during the last four weeks of life [11]. In the present ICI group, the rate of oncological treatment during the last four weeks of life was numerically lower (19%), although sequential studies from different time periods and inter-study comparisons are hampered by sources of bias. It was also reported that 53% of the patients from our earlier non-ICI cohort died in the hospital [12]. This rate was lower in the present non-ICI group (34%), but comparably high in the ICI group (59%). As mentioned previously, this difference might be the result of thoracic symptoms that had to be addressed with interventions reducing pleural effusions. Possibly, these patients had palliative care needs that were difficult to solve outside of a hospital. Our standard clinical pathways did not include early palliative care, a strategy that reduces aggressive EOL care and hospitalization [16]. The involvement of the palliative care team often started relatively late after diagnosis. Between 50 and 59% of the present patients had contact with the palliative care team during the disease trajectory.

Muchnik et al. evaluated 75 patients who were 70 years or older with advanced-stage NSCLC treated with an ICI between 2015 and 2017 [17]. Of these, 49% had ECOG PS ≥2 disease. Median survival for the whole cohort was 8.2 months. No ICI-related deaths were observed. Hospitalizations during ICI treatment occurred in 72% of the cases. Toxicity generally did not differ by age, comorbidity, or PS. The relatively high rates of hospitalization during ICI treatment in this study highlight the vulnerability of older adults with advanced NSCLC. Also, in our study, hospitalization during the last three months of life was common. There are reasons to believe that hospitalization and hospital death in the ICI group were mainly related to different disease characteristics and not to ICI administration itself. This would also be expected based on the toxicity results of the seminal prospective clinical studies [3-6]. However, real-world data from elderly rural patient cohorts might differ from those obtained in clinical trials. It is therefore necessary to perform additional and larger studies about the ICI-related patterns of terminal care, taking into account the fact that resource use varies between countries [18].

## Conclusions

This analysis did not identify any concerns regarding fatal toxicity of ICI treatment during initial implementation. However, hospital death was more common after ICI therapy and the patients spent more days in the hospital during the three months before death. Due to several different baseline parameters, we can reasonably conclude that hospitalization and hospital death in the ICI group were mainly related to unevenly distributed disease characteristics and not to ICI administration itself. Since real-world data from rural patient cohorts might differ from those obtained in clinical trials, it is necessary to perform additional and larger studies about the ICI-associated patterns of palliative interventions, terminal care, and hospital deaths.
